# Dengue-specific subviral nanoparticles: design, creation and characterization

**DOI:** 10.1186/1477-3155-11-15

**Published:** 2013-05-25

**Authors:** Niyati Khetarpal, Ankur Poddar, Satish K Nemani, Nisha Dhar, Aravind Patil, Priyanka Negi, Ashiya Perween, Ramaswamy Viswanathan, Heinrich Lünsdorf, Poornima Tyagi, Rajendra Raut, Upasana Arora, Swatantra K Jain, Ursula Rinas, Sathyamangalam Swaminathan, Navin Khanna

**Affiliations:** 1 Recombinant Gene Products Group, International Centre for Genetic Engineering & Biotechnology, Aruna Asaf Ali Marg, New Delhi, 110067, India; 2 Leibniz University of Hannover, Technical Chemistry-Life Science, Hannover, Germany; 3 Helmholtz Centre for Infection Research, Braunschweig, Germany; 4 Department of Biotechnology, Jamia Hamdard, Hamdard Nagar, New Delhi, 110062, India

**Keywords:** Dengue envelope domain III, Hepatitis B surface antigen, Virus-like particle, Bionanoparticles, Pichia pastoris

## Abstract

**Background:**

Dengue is today the most significant of arboviral diseases. Novel tools are necessary to effectively address the problem of dengue. Virus-like particles (VLP) offer a versatile nanoscale platform for developing tools with potential biomedical applications. From the perspective of a potentially useful dengue-specific tool, the dengue virus envelope protein domain III (EDIII), endowed with serotype-specificity, host receptor recognition and the capacity to elicit virus-neutralizing antibodies, is an attractive candidate.

**Methods:**

We have developed a strategy to co-express and co-purify Hepatitis B virus surface (S) antigen in two forms: independently and as a fusion with EDIII. We characterized these physically and functionally.

**Results:**

The two forms of the S antigen associate into VLPs. The ability of these to display EDIII in a functionally accessible manner is dependent upon the relative levels of the two forms of the S antigen. Mosaic VLPs containing the fused and un-fused components in 1:4 ratio displayed maximal functional competence.

**Conclusions:**

VLPs armed with EDIII may be potentially useful in diagnostic, therapeutic and prophylactic applications.

## Background

Currently, dengue represents the most important arboviral disease that places nearly half the global population at risk [1]. The mosquito-borne disease is caused by four closely related, yet antigenically distinct, serotypes of dengue viruses (DENV-1, -2, -3 and −4) [2]. All four DENVs and their mosquito vectors are co-prevalent in more than one hundred tropical/sub-tropical countries. Each of the DENVs can cause disease ranging from mild dengue fever to severe dengue hemorrhagic fever and potentially fatal dengue shock syndrome [3]. Tools for diagnosing, treating and ultimately preventing dengue are urgently needed [4]. While increasingly reliable diagnostic tools are becoming available [4-6], antivirals [4,7,8] and vaccines [9,10] for dengue continue to be elusive.

Nanobiotechnology, which seeks to use naturally occurring as well as engineered nanoscale biomaterials to make functional systems, is rapidly emerging as a platform for the development of novel nanotools with potential biomedical applications [11-13]. We are interested in exploring potential applications of this technology to infectious diseases. We have utilized Eu^3+^-doped polystyrene nanoparticles as very sensitive reporters for detecting Hepatitis B virus [14] and human immunodeficiency virus [15] infections. We have also used nanoparticles of biological origin, namely, virus-like particles (VLPs). Many viral capsid proteins possess intrinsic ability to self-assemble into VLPs when expressed in recombinant insect, yeast and mammalian host systems [13]. Recently, we have begun exploring VLP platforms for the display of foreign antigens [16]. The foreign antigen we focus on is derived from the major DENV structural antigen on the virion surface, the envelope protein. Multiple antigenic determinants that are largely serotype-specific map to a C-terminal ~100 amino acid (aa) region of this protein [17]. Further these antigenic determinants tend to elicit DENV-neutralizing antibodies and coincidentally, this C-terminal region, which is known as envelope domain III (EDIII), is also implicated in host receptor recognition [18]. For these reasons, we believe EDIII offers an attractive precursor for developing nanoparticle tools that may be useful in addressing the problem of dengue.

Recombinant Hepatitis B virus surface (S) antigen is well-documented to self-assemble into 20–22 nm VLPs [19-21] and is the main component of commercial Hepatitis B vaccines [22,23]. In this study, we sought to exploit the S antigen VLP as a carrier for DENV-2 EDIII. To this end we fused EDIII to the amino-terminus of S to create a fusion antigen, herein referred to as ES antigen. We used the methylotrophic yeast *Pichia pastoris*, which we have found earlier to express S antigen VLPs to very high levels [20], to express the ES antigen. We found that for DENV-2 EDIII to be displayed on the VLP surface in way that made it accessible to DENV-2-specific antibody and to the host cell receptor, it was necessary to co-express the ES fusion antigen with un-fused S antigen. We describe our strategy to co-express ES and S antigens in *P. pastoris*, their co-purification and structural as well as functional characterization of the resultant mosaic ES,S VLPs.

## Results

### Strategy to co-express *ES* fusion antigen gene in the background of 0–4 copies of *S* gene

**Figure 1:**
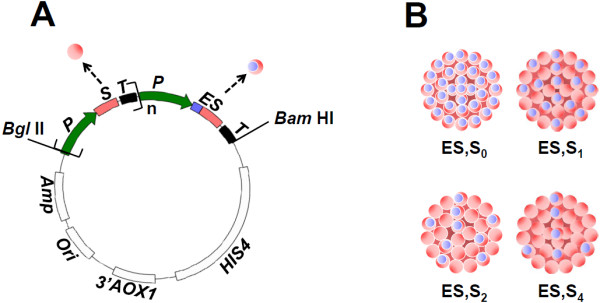
**Strategy to produce DENV-2 EDIII containing VLPs in *P. pastoris*.** (**A**) Map of pAO815-based *P. pastoris*-integrative expression vector carrying HBsAg (*S*) and EDIII-2-HBsAg (*ES*) antigen gene expression cassettes in tandem. Each gene is present in an independent expression cassette with the *AOX1* promoter (*P*) on the 5’ side and the *AOX1* transcription terminator (*T*) on the 3’ side. A family of vectors was created in which the number (n) of *S* gene expression cassettes varied from 0–4. Other elements present in the vector include the ampicillin resistance marker (*Amp*), the bacterial replication origin (*Ori*), 3’ terminal sequences of the *AOX1* gene (*3’AOX1*) and the yeast histidinol dehydrogenase marker gene (*HIS4*). The dashed arrows point to symbols used to depict the proteins encoded by the ‘*S*’ (red sphere) and ‘*ES*’ (red and blue sphere) genes. Unique sites used for cassette assembly are shown. (**B**) Schematic representations of the putative VLPs predicted to be generated from the construct when the ‘S’ antigen copy number is 0 (ES,S_0_), 1 (ES,S_1_), 2 (ES,S_2_) or 4 (ES,S_4_) per copy of the ‘ES’ fusion antigen.

The ES fusion antigen was designed to contain the DENV-2 EDIII (spanning aa residues 297–400 of the full-length envelope protein), linked in-frame to the amino-terminus of the Hepatitis B S antigen through a pentapeptide linker (Additional file 1: Figure S1). The *ES* gene was placed under the transcriptional control of the *P. pastoris* alcohol oxidase 1 (*AOX1*) promoter. This construct is designed to express a ~37 kDa ES antigen with the first 104 and the last 226 aa residues representing, respectively, the DENV-2 EDIII and S fusion partners, in the absence of any un-fused S antigen. As the ES antigen by itself did not form VLPs efficiently (see below), we attempted to co-express varying levels of un-fused S antigen as well. For this, 1, 2 and 4 copies of an *S* gene expression cassette (once again, *AOX1* promoter-driven), were inserted at the 5’ end of the ES expression cassette in a sequential head-to-tail manner, using an *in vitro* multimerization strategy described previously [19]. The S antigen expression cassette is designed to generate a ~24 kDa protein, whose aa sequence is identical to the C-terminal 226 aa residues of the ES antigen. A schematic representation of the basic map of these constructs is depicted in Figure 1A. Putative VLPs that could arise out of ES antigen in the absence of any S antigen co-expression (designated ES,S_0_), and in the presence of 1, 2 and 4 copies of the S antigen, designated as ES,S_1,_ ES,S_2_ and ES,S_4,_ respectively, are shown schematically in Figure 1B.

### Creation of *P. pastoris* clones designed to co-express ES,S_0-4_ antigens

**Figure 2:**
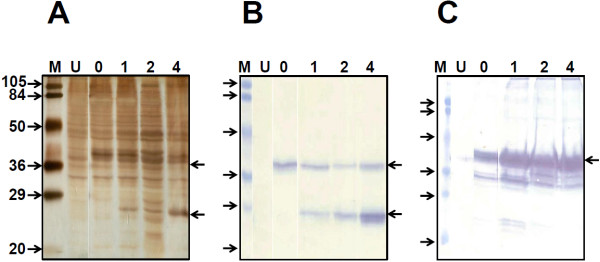
**Co-expression of ES fusion antigen with 0–4 copies of S antigen in *P. pastoris*.** (**A**) Methanol-induced *P. pastoris* clones harboring the *ES* fusion gene in the background of 0, 1, 2 and 4 copies of the *S* gene were lysed and run on SDS-PAGE (lanes are indicated by the *S* gene copy number), followed by silver staining. An un-induced sample (of clone ES,S_4_) was analysed in lane ‘U’. (**B**) The same samples as in ‘A’ were transferred onto a nitrocellulose membrane after SDS-PAGE, and subjected to immunoblotting with mAb 5S. (**C**) Immunoblot analysis similar to that shown in panel ‘B’, but probed using mAb 24A12. In all three panels, pre-stained protein markers were run in lanes ‘M’; their sizes in kDa are shown to the left of panel ‘A’. The arrows to the right indicate the positions of the ES fusion (upper) and the S (lower) antigens.

The *ES* gene fusion constructs in the background of 0–4 copies of *S* gene, described above, were integrated into *P. pastoris* using standard methods. Selected transformants representing each of the constructs were analysed for ES and S antigen expression upon methanol induction. The results of a typical experiment analysing the polypeptide profiles of induced cell lysates are shown in Figure 2A. Upon induction, a new polypeptide band of ~24 kDa, consistent with the predicted size of the S antigen, is apparent in all clones harboring the *S* gene (compare lanes ‘1-4’ with lane ‘U’). As expected, the clone lacking the *S* gene did not express the 24 kDa band (compare lanes ‘U’ with ‘0’). Interestingly, the band intensity of this ~24 kDa polypeptide seemed to reflect the *S* gene copy number, being indiscernible in the *S* gene-lacking clone (lane ‘0’), but maximal in the clone harbouring 4 copies of the *S* gene (lane ‘4’). However, it was difficult to unambiguously detect the presence of a ~37 kDa protein band, the predicted size of the ES antigen, in these clones. To confirm if indeed the ES antigen is expressed by these clones we carried out an immunoblot assay using an in-house S antigen-specific mAb 5S, which is expected to recognize both ES as well as S antigen polypeptides. The data unambiguously revealed the presence of ~37 kDa ES antigen in the induced lysates of all four clones (Figure 2B). As expected three of these clones also co-expressed the ~24 kDa S antigen. Interestingly, once again, the levels of S antigen manifested a clearly visible copy number effect. A densitometric analysis of the relative intensities of the ~24 kDa bands in the 1, 2 and 4 copy clones (Figure 2B, lanes ‘1’, ‘2’ and ‘4’) was found to be 1, 1.8, and 3.9, respectively, closely matching the corresponding *S* gene copy number for these three clones. This copy number effect is consistent with our earlier observation which showed that increasing *S* gene copy number is paralleled by a corresponding increase in S mRNA and protein levels [19]. Further, a densitometric comparison of ES and S antigen expression levels also showed that the S/ES ratios were 0.8, 2.4 and 3.7, respectively, for ES,S_1_, ES,S_2_ and ES,S_4_, This is in good agreement with the predicted ratios of 1, 2 and 4, for ES,S_1_, ES,S_2_ and ES,S_4_, respectively. Performing the immunoblot using an in-house EDIII-specific mAb 24A12 recognized only the ~37 kDa band as expected (Figure 2C). The observed immunoreactivities of the ~37 and ~24 kDa polypeptides are consistent with those of ES and S antigens, respectively. Collectively, these data confirmed that our panel of *P. pastoris* clones co-express ES and S antigen, the latter at calibrated levels through pre-determined gene copy number establishment. It is to be noted that the constructs and *P. pastoris* clones described in this manuscript are based on DENV-2 EDIII. We have extended this approach to the EDIIIs of the remaining three DENV serotypes as well and generated corresponding *P. pastoris* clones (data not shown).

### The ES,S_0-4_ antigens are predominantly associated with the P fraction of induced cell lysates

**Figure 3:**
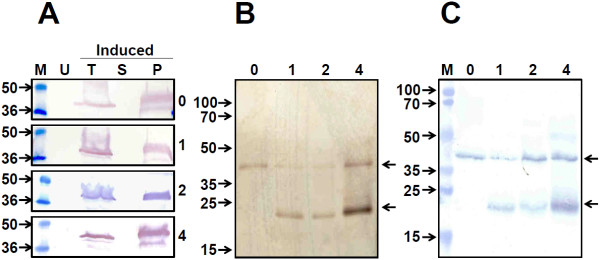
**Purification of the *P. pastoris*-expressed ES,S**_**0**_**, ES,S**_**1**_**, ES,S**_**2**_**, and ES,S**_**4**_**, antigens.** (**A**) Localization of the recombinant ES fusion antigens by Western blot analysis using mAb 24A12. Four ES fusion protein-expressing *P. pastoris* clones, co-expressing 0, 1, 2 and 4 copies of the S antigen (indicated by Arabic numerals on the right side of the immunoblot strips) were analysed. Aliquots of *P. pastoris* cultures were analysed before (U) and after methanol induction; induced cell extracts were analysed before (T) and after separation into supernatant (S) and pellet (P) fractions. Pre-stained protein markers were analysed in lanes ‘M’. Their sizes (in kDa) are indicated to the left. (**B**) Polypeptide profiles of the recombinant ES,S_0_ (lane ‘0’), ES,S_1_ (lane ‘1’), ES,S_2_ (lane ‘2’), and ES,S_4_ (lane ‘4’), antigens purified from the ‘P’ fractions of the induced cells (described in panel ‘**A**’), visualized by silver staining. (**C**) Western blot analysis of the purified proteins shown in panel ‘**B**’, using mAb 5S. Pre-stained protein markers were run in lane ‘M’. All other lanes are similar to panel ‘**B**’. For panels ‘**B**’ and ‘**C**’, the arrows on the left indicate the positions and sizes (in kDa) of the protein markers analysed in parallel; the arrows to the right indicate the positions of the ES (upper) and S (lower) antigens.

It has been reported that the S antigen expressed in yeast associates closely with the cellular membrane components [21,24,25]. This would predict that the ES antigen may also manifest this tendency. To investigate this, we analysed the distribution of the ES antigen between the soluble and membrane-enriched P fractions of induced cell lysates. A typical localization experiment is shown in Figure 3A. Induced total cell lysates (T) were separated into supernatant (S) and pellet (P) fractions, and all three samples of each induced clone were evaluated by immunoblot assay using mAb 24A12. This revealed that regardless of S antigen co-expression, the ES antigen was almost exclusively associated with the P fraction. We next sought to quantify the levels of the recombinant antigens in the P fraction using a sandwich ELISA approach. Antigens were captured using either EDIII-specific or S-specific mAb and revealed using a second S antigen-specific mAb conjugate (Additional file 1: Figure S2). This experiment confirmed the association of both ES and S antigens almost exclusively with the P fraction for all clones. In line with expectation, when recombinant antigens were captured using mAb specific to S antigen, we observed a copy number-dependent increase in antigen levels. However, it is to be noted that this ELISA recognizes both ES and S antigens. Interestingly, even though all *P*. *pastoris* clones were designed to express ES antigen from a single copy gene, we found ES levels paralleled the increase in S antigen gene copy number, when mAb 24A12 was used for capture. We interpret this data to reflect more efficient capture of ES antigen in the sandwich ELISA as the levels of co-expressed S antigen increases.

### Co-purification of the ES and S antigens from induced *P. pastoris* cells

As a prelude to purification, we sought to optimize the conditions of induction. To this end, methanol concentration and induction duration were varied with respect to each other, followed by analysis of ES antigen in the P fractions by immunoblotting and ELISA. Based on these results (Additional file 1: Figure S3), we carried out induction at 1% methanol for 3 days. As the ES antigen was predominantly associated with the P fraction, we decided to adapt a protocol recently developed in our laboratory that exploits a similar behaviour of the S antigen for its purification [21]. Thus, the P fraction from induced cells which served as the starting material was solubilized in the presence of detergent and urea, subjected to polyethylene glycol 6000 (PEG 6000) precipitation, tangential flow filtration (TFF) using a 300 kDa cut-off membrane, and finally chromatography on Phenyl Sepharose. The peak fractions were pooled and aliquots analysed by silver stained SDS-PAGE as shown in Figure 3B. The data reveal that both the ES and S antigens expressed by each of the ES,S_1-4_ clones co-purified through the multistep purification protocol. A densitometric scan revealed >95% purity for each. That the co-purified proteins were indeed ES and S was confirmed by their identification in a Western blot analysis using mAbs 5S (Figure 3C) and 24A12 (*data not shown*). Importantly, both the silver stained gel and the immunoblot analyses revealed the copy number effect in S antigen levels. To explore if purification could be achieved in the absence of urea, we used an alternate protocol in which the starting material was the soluble fraction obtained from total cell lysate prepared in the presence of 0.6% Tween 20 [25]. This resulted in low yields (data not shown), commensurate with the low concentrations in the S fractions (Additional file 1: Figure S2).

### Co-expressed ES and S antigens form VLPs

**Figure 4:**
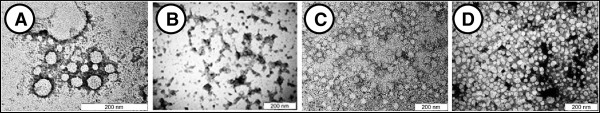
**Electron microscopic visualization of purified ES,S antigens.** The purified ES,S_0_ (panel ‘**A**’), ES,S_1_ (panel ‘**B**’), ES,S_2_ (panel ‘**C**’), and ES,S_4_ (panel ‘**D**’), antigens shown in Figure 3B were stained with uranyl acetate and visualized by electron microscopy.

The demonstration that ES and S antigens could be co-purified, particularly in a protocol that involved TFF across a 300 kDa cut-off membrane suggested that the ES and S antigens are closely associated with each other and presumably incorporated into higher order structures. To investigate this further, we analysed a partially purified preparation (obtained after the TFF step) from the ES,S_4_ clone on a CsCl density gradient. Fractions were analysed in ES- and S-specific sandwich ELISAs, referred to above. This revealed that both ES and S antigens co-sedimented together in the bottom one-third of the gradient. An aliquot of this fraction containing both ES and S peaks by electron microscopy revealed the presence of discrete VLPs (Additional file 1: Figure S4). To answer the question if VLP formation occurs when the levels of S antigen co-expression was lower, aliquots of the pooled peak phenyl sepharose fractions from each of the four preps was analysed by electron microscopy (EM) as shown in Figure 4. This study revealed that the ES,S_0_ antigen preparation appeared to contain large polydisperse aggregates without any discrete VLPs. Given that the S antigen expressed in *P. pastoris* forms VLPs efficiently [20,21], this observation may likely indicate that the EDIII fusion partner in the ES antigen hinders efficient VLP formation. However, the ability to form VLPs was visibly enhanced with increasing levels of co-expressed S antigen. Thus, the VLPs in the ES,S_4_ prep manifested the presence of well-formed VLPs with the highest degree of homogeneity. Taken together, the purification and EM data show that the ES,S_0_ represents aggregates containing only the ES fusion antigen, whereas the ES,S_1_, ES,S_2_ and ES,S_4_ represent mosaic VLPs containing progressively increasing proportions of S antigens as well. Further, the incorporation of ES into VLP is progressively improved by increasing the relative proportion of S antigen. Similar VLP formation was evident when purification was done in the absence of urea, except that yields were extremely low (data not shown).

### S antigen content of VLPs contributes to optimal EDIII accessibility

**Figure 5:**
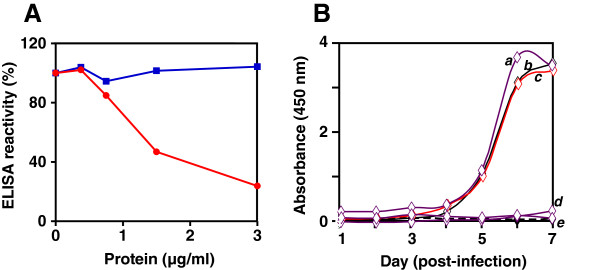
**Functional characterization of the ES,S VLPs.** (**A**) Competitive ELISA analysis. Anti-EDIII mAb was pre-incubated either with S (blue curve) or ES,S_4_ (red curve) antigen VLPs, followed by titration of residual mAb reactivity on yeast-expressed EDIII-2 antigen-coated ELISA plates. (**B**) Binding blocking analysis. Vero cells were pre-incubated with S (curve ‘c’), ES,S_0_ (curve ‘a’), ES,S_2_ (curve ‘d’) and ES,S_4_ (curve ‘e’) VLPs and then infected with DENV-2. Control DENV-2 infection in the absence of any VLP pre-incubation (curve ‘b’) and mock-infections (dashed curve) were carried out in parallel. The progress of infection was monitored over a week by measuring the release of NS1 antigen in the culture supernatant. Experiments in both panels **A** and **B** were repeated twice; results of one representative experiment each are shown.

Our objective was to display functionally competent DENV EDIII moieties on the VLP surface. Only then would such VLPs have potential utility for developing customized dengue-specific nanotools. To assess surface accessibility of EDIII, we employed a competitive ELISA wherein we measured the capacity of these VLPs to compete with free monomeric EDIII antigen for specific antigen-binding sites on anti-EDIII antibodies. In the experiment shown in Figure 5A, pre-incubation of the EDIII-specific mAb 24A12 with S antigen (blue curve) did not adversely affect its ELISA reactivity towards yeast-expressed DENV-2 EDIII-2 [26]. On the other hand, pre-incubation with the ES,S_4_ VLPs (red curve) produced a dose-dependent reduction in the capacity of the mAb towards recombinant EDIII-2, resulting in 50% reduction at a concentration of 1.4 μg/ml. The ELISA reactivity of this mAb upon pre-incubation with ES,S_0_, ES,S_1_ and ES,S_2_ VLPs displayed profiles lying between these two extremes (*data not shown*). That all these VLPs did compete, albeit at different efficiencies, is clearly a reflection of the surface accessibility of the VLP-displayed EDIII. It is noteworthy that the relative proportion of the EDIII moiety is highest in ES,S_0_ and the lowest in ES,S_4_ VLPs. That ES,S_4_ despite its lowest EDIII moiety content manifests the highest efficiency in competing for mAb binding suggests that it provides for maximal ease of accessibility, and therefore, display of EDIII moieties, on the VLP surface. It automatically follows that, if EDIII is displayed on the VLP surface, it must be able to compete with infectious DENV-2 for host cell receptor-binding. To this end, we exposed Vero cell monolayers to the different VLP preparations followed by DENV-2 infection. Successful infection would be followed by viral replication within the cells. We followed this using viral NS1 antigen secretion in the culture supernatant as shown in Figure 5B. The data showed that the ES,S_2_ and ES,S_4_ VLPs were able to potently inhibit NS1 antigen secretion in contrast to the ES,S_0_, and ES,S_1_ VLPs, which were virtually ineffective in this regard. Once again, the inverse relationship between relative EDIII content and its functional competence of the VLPs was reflected in this experiment.

Taken together, the competitive ELISA and virus binding-blocking experiments support the notion that mosaic VLPs containing the ES and S antigens in 1:4 ratios display EDIII on the surface in a manner that is compatible with its optimal interaction with antibodies and the host cell receptors.

## Discussion

The magnitude of dengue as a public health problem is accentuated by the lack of drugs and vaccines [3,4,9]. The emergence of nanotechnology and its increased recent focus on bionanomaterials has opened up new interdisciplinary avenues exploring novel biomedical applications [11-13]. Our interest is in exploring this area with the ultimate goal of developing nanotools that could help address the dengue problem. In this context, our interest is in the use of bionanomaterials which have the intrinsic advantages of biocompatibility and biodegradability [12]. In contrast to naturally occurring viral nanoparticles, which are biohazardous, their genome-free counterparts, the VLPs, are being increasingly preferred for developing biomedical nanotools [11-13]*.*

The S antigen of Hepatitis B represents the classic example of a viral protein which can independently assemble into nanoparticles [19-21] and is the basis of a highly successful VLP vaccine [22,23]. We have sought to endow DENV specificity to these S VLPs, as a first step towards developing bionanoparticles with potential dengue-specific applications. To confer DENV specificity we chose a ~100 aa domain known as EDIII, for reasons mentioned already, and genetically linked it to the N-terminus of the S antigen, and expressed it using *P. pastoris*. We observed that the resultant ES antigen did not form VLPs efficiently. Moreover, it did not display EDIII optimally based on specific assays to test its functionality. In order to obtain VLPs with functionally competent EDIII, we developed a strategy to co-express un-fused S antigen at calibrated levels using defined *S* gene copy numbers. The resultant mosaic VLPs contain two components, ES antigen and un-fused S antigen. Both ES and S antigens tend to be membrane associated, by virtue of the latter’s intrinsic hydrophobicity [21,24,25]. This necessitated their co-purification starting from the membrane fraction using a protocol we developed recently for S antigen purification [21]. A noteworthy feature of this purification scheme is that it eliminates CsCl centrifugation, a bottleneck in downstream processing. Based on a variety of criteria including co-purification through 300 kDa cut-off TFF, CsCl gradient sedimentation analysis, EM and sandwich ELISA’s, we found that ES and S antigens associate together to form mosaic VLPs. Using competitive ELISA and bind blocking assays, we demonstrated that mosaic VLPs containing 4 copies of S antigen per copy of the ES antigen displayed EDIII moiety optimally.

The data have significant implications from the perspective of dengue-specific nanoparticulate tools. As EDIII can induce virus-neutralizing antibodies [26,27], the repeat-pattern architecture of the mosaic VLP can potentially augment its vaccine potential. This notion is backed by the fact that vaccines based on the VLP platform are already available for Hepatitis B and human papilloma virus infections [13]. Strategies to load VLPs with drugs and target viral nanoparticles to tumors are being actively investigated [12]. Thus, if one can envisage charging these mosaic VLPs with a drug against dengue, these VLPs through their surface displayed EDIII can be targeted for drug delivery to DENV-susceptible cells through host receptor recognition. For vaccine and drug-delivery applications, the non-replicating, infectious viral genome-free VLPs offer the advantage of in-built safety. Additionally, as the EDIII is useful in serotype identification [27], these mosaic VLPs could also serve in diagnostic serotyping of DENV infections.

## Conclusions

This work shows that DENV EDIII fused to the S antigen, co-expressed with un-fused S antigen forms mosaic VLPs. VLPs containing the fused and un-fused components in 1:4 ratio displayed EDIII optimally. These EDIII-displaying VLPs have potential vaccine, drug-delivery and diagnostic applications. As these VLPs can be produced in *P. pastoris*, which is capable of high productivity in simple media, they can be expected to be inexpensive, a significant advantage for resource-poor regions where dengue is endemic. Finally, the approach developed here could serve as the basis for a common bionanoparticle platform for other infections as well.

## Methods

The genes *ES* (1 kb) and *S* (0.7 kb), codon-optimized for *P. pastoris* expression were synthesized by Geneart AG (Regensburg, Germany). DENV-2 (NGC strain) stock was from previously reported work [28]. DENV-2 EDIII-specific monoclonal antibody (mAb) 24A12 [26] and S antigen-specific mAb 5S [21] were in-house reagents.

A panel of four ES (1 copy) expression vectors, co-expressing 0, 1, 2 and 4 copies of the S antigen was created using a head-to-tail *in vitro* multimerization method [19]. The *ES* gene was cloned into the unique *Eco RI* site of pAO815 to generate the expression plasmid pAO-ES,S_0_. To provide for co-expression of S antigen, 1, 2 and 4 copies of an *S* gene expression cassette were inserted sequentially to generate pAO-ES,S_1_, pAO-ES,S_2_ and pAO-ES,S_4_, respectively. Each of the four constructs above was integrated into the genome of *P. pastoris* (GS115) as described before [19].

Typically, yeast cultures were grown at 30°C to log phase in buffered glycerol-containing medium (BMGY) and switched to buffered 1% methanol-containing medium (BMMY) for induction for 72 hours. Total (T) lysates for analytical experiments were prepared from methanol-induced cells using glass beads in a detergent-containing buffer as described [21]. A portion of the total lysate was separated by centrifugation into S- and the membrane-enriched P-fractions. Suitable dilutions of T, S and P (urea-solubilized) fractions in urea-free buffer were used for sandwich ELISA and immunoblotting experiments.

For purification, we followed a method reported recently for *P. pastoris*-expressed S antigen purification starting from the membrane fraction [21] with some modifications as follows. Induced biomass (100 grams wet weight) was lysed with glass beads (5 cycles) in a Dyno-mill (WAB, Muttenz, Switzerland) and centrifuged to separate out the membrane-enriched P fraction, which was solubilized in a buffer containing 4 M urea and 2% Tween 20. The solubilized P fraction was clarified and subjected to 5% PEG 6000 precipitation overnight. The post PEG supernatant was clarified by centrifugation and 0.45 μ filtration and subjected to TFF across a 300 kDa membrane using 12 L of TFF buffer with step-wise reduction in urea (4 M to 0 M). The TFF retentate was chromatographed on Phenyl 600 M Toyopearl resin (Tosoh Bioscience, Stuttgart, Germany). Bound proteins were eluted using a 0-8 M urea step gradient (with 2 M increase at each step lasting 5 bed volumes) in 20 mM sodium bicarbonate buffer (pH 9.6). Column fractions were analysed by SDS-PAGE, purified peak fractions pooled, and dialyzed against 1× PBS. In some instances, we also carried out purification from the S fraction in the absence of urea as described [25].

Proteins were characterized by ELISA, immunoblotting, CsCl gradient centrifugation and EM. ES protein was detected by sandwich ELISA using two formats. In one case, DENV-2 EDIII-specific mAb 24A12 was used for antigen capture, followed by revealing it with an S antigen-specific mAb-enzyme conjugate. In the second case, both capture and reveal mAbs were S antigen-specific (from Hepanostika Ultra kit, Biomerieux, Marcy L’Etoile, France). Immunoblot analyses were performed essentially as reported earlier [16,21] using either mAb 24A12 or 5S mAb. Densitometric analysis of the resultant blots was performed using freely available image analysis software from NIH (ImageJ software). CsCl gradient fractionation was performed as described previously [19]. The presence of VLPs in the purified preparations was visualized by EM as before [16].

The functional status of EDIII moiety on the VLPs was assessed by its ability to (i) compete for binding to a specific mAb and (ii) inhibit DENV-2 binding to host cell surface receptors. Competitive ELISA was done essentially as reported [16] using mAb 24A12. Binding blocking assay was performed using Vero cells (American Type Culture Collection, Virginia, USA). Briefly, cells were pre-exposed separately to each of the VLP preparations for 1 hour followed by infection with DENV-2. Culture supernatants were sampled at daily intervals for release of viral NS1 antigen using Dengue NS1 Platelia kit (BioRad Inc., USA) as per the manufacturer’s directions [29].

## Abbreviations

AOX1: Alcohol oxidase 1; DENV: Dengue virus; EDIII: Envelope domain III; ES: EDIII of DENV-2 fused to Hepatitis B virus surface antigen; ES,S0-4: ES antigen co-expressed with 0–4 copies of Hepatitis B virus surface antigen; PEG: Polyethylene glycol; S: Surface antigen of Hepatitis B virus; S fraction: Supernatant fraction; P fraction: Pellet fraction; TFF: Tangential flow filtration; VLP: Virus-like particle.

## Competing interests

The authors declare that they have no competing interests.

## Authors’ contributions

Conceived and designed the study: SS and NaK; plasmid construction and integration into *P. pastoris*: NiK, AnP, SKN, ND, PN; expression optimization and purification: NiK, AnP, SKN, ArP, AsP and RV; EM analysis: PT and HL; Functional characterization: NiK, AnP, SKN, UA and RR; data interpretation: SKJ, UR, SS, NaK; wrote the manuscript: UR, SS and NaK; all authors read and approved the manuscript.

## Supplementary Material

Additional file 1 Word file containing more experimental details and supplementary data (Figures S1-S4). Click here for file
