# Supplementary material for: Dengue-specific subviral nanoparticles: design, creation and characterization
Source: J Nanobiotechnology. 2013 May 25;11:15. doi: 10.1186/1477-3155-11-15 (PMC3680219; doi:10.1186/1477-3155-11-15)
Supplement: Additional file 1 — Word file containing more experimental details and supplementary data (Figures S1-S4). [file 1477-3155-11-15-S1.docx]

**SUPPLEMENTARY MATERIAL**

**1. EXPERIMENTAL DETAILS**

**1.1 Materials**

The genes *ES* (1 kb) and *S* (0.7 kb), codon-optimized for *P. pastoris* expression were synthesized by Geneart AG (Regensburg, Germany). *E. coli* (strain DH5α), *P. pastoris* (strain GS115) and the *P. pastoris* integrative plasmid pAO815 were purchased from Invitrogen Life Technologies (Carlsbad, USA). Plasmid pAO815 contains the methanol-inducible Alcohol Oxidase 1 (*AOX1*) promoter, a cloning site and sequences essential for integration into the host *AOX1* locus. It also carries ampicillin resistance and *HIS4* markers for selection in *E. coli* and *P. pastoris*, respectively. Vero cells were from American Type Culture Collection (Virginia, USA). DENV-2 (NGC strain) stock was from previously reported work (*Khanam et al, 2009*).

Phenyl 600M Toyopearl was from Tosoh Bioscience (Stuttgart, Germany). Pellicon-2 polyethersulphone membrane (300 kD cut-off) was from Millipore (Billerica, USA). DENV-2 EDIII-specific monoclonal antibody (mAb) 24A12 (*Batra et al, 2010*) and S antigen-specific mAb 5S (*Patil & Khanna, 2012*) were in-house reagents. Anti-mouse IgG antibody-horseradish peroxidase (HRPO) conjugate was from Calbiochem, CA, USA. The HRPO substrate 3, 3’, 5, 5’-Tetramethylbenzidine (TMB-soluble & -insoluble) and acid-washed glass beads (425-600 microns) were from Sigma-Aldrich, MO, USA. The Hepanostika Ultra kit for S antigen ELISA was from Biomerieux (Marcy L’Etoile, France). The Platelia Dengue NS1 ELISA kit was from BioRad Inc., USA. BCA protein assay reagent was from Thermo Scientific, Rockford, USA.

**1.2 Creation of ES,S co-expression clones**

A panel of four ES (1 copy) expression vectors, co-expressing 0, 1, 2 and 4 copies of the S antigen was created using a head-to-tail *in vitro* multimerization method (*Vassileva et al, 2001*). The *ES* gene was cloned into the unique *Eco RI* site of pAO815 to generate the expression plasmid pAO-ES,S_0_. In this construct, the *ES* gene is placed between the *AOX1* promoter on the 5’ side and the alcohol oxidase terminator on the 3’ side. The 5’ and 3’ ends of the *ES* gene expression cassette are flanked by *Bgl* II and *Bam* HI restriction sites, respectively. This construct is designed to express only the ES antigen. To provide for co-expression of S antigen, an *S* gene expression cassette (constructed using a similar strategy) was inserted as a *Bgl* II/*Bam* HI fragment into the *Bam* HI site of pAO-ES,S_0_ resulting in pAO-ES,S_1_, a vector carrying one *ES* gene expression cassette and one *S* gene expression cassette in tandem. Extending this approach, we created plasmids pAO-ES,S_2_ and pAO-ES,S_4_, containing 2 and 4 tandem repeats of the *S* gene expression cassette (besides the single *ES* gene expression cassette). Each of the four constructs above was integrated into the genome of *P. pastoris* (GS115) by electroporation followed by selection in the absence of histidine in the medium (*Vassileva et al, 2001*). The resultant *P. pastoris* transformants were screened for ES antigen expression by Western blotting (see below) using EDIII-specific mAb 24A12.

**1.3 Induction and extraction**

Typically, yeast cultures were grown at 30^o^C to log phase in buffered glycerol-containing medium (BMGY) and switched to buffered 1% methanol-containing medium (BMMY) for induction. In preliminary experiments, methanol concentrations ranging from 0.5-3%, and induction times from 6-96 hours were tested. Based on this, routine induction was usually with 1% methanol maintained for 72 hours.

Extracts for analytical experiments were prepared essentially as described earlier (*Patil & Khanna, 2012*). Induced cells (equivalent to 100 OD) were suspended in 500µl cell suspension buffer, CSB [20mM Phosphate (pH7.2)/150mM NaCl/8% glycerol/5mM EDTA/0.5% TritonX-100]. This was mixed with 500µl glass beads pre-washed with CSB and extracted overnight on a thermomixer set to 1400 rpm at 4^o^C. The lysate was collected. The beads were washed with 500µl CSB and pooled with the lysate to obtain ~1ml of total lysate (T). A portion of the total lysate (typically 500µl) was spun down in the microfuge at max speed (4^o^C) and separated into supernatant (S) and the membrane-enriched pellet (P) fractions. The latter fraction was solubilized by extracting with 500µl 1× phosphate buffered saline (PBS)/8M urea/2% Tween 20 (on a thermomixer at 25^o^C for 4 hours at 1400 rpm). This was spun down to obtained solubilized, clarified P fraction. The T, S and P fractions were analysed by sandwich ELISA and immunoblotting (see below).

**1.4 Purification**

Overnight starter culture of each *P. pastoris* clone was inoculated into 4L BMGY in multiple baffled flasks (occupying 20-25% flask volume to permit adequate aeration) and grown (30^o^C/250 rpm) to log phase (OD_600_=25-30). Cells were collected by centrifugation at room temperature (RT), washed with sterile 1× PBS and re-suspended in half the starting volume of 1% methanol-containing BMMY. Induction was maintained for 72 hours with methanol addition every 12 hours. At the end of induction time, cells were pelleted down, washed twice with sterile 1× PBS and stored at -70^o^C until extraction.

One hundred grams of induced biomass were suspended in 400ml CSB and lysed with glass beads (5 cycles) in a Dyno-mill (WAB, Muttenz, Switzerland). The resultant lysate was centrifuged at 10,000 rpm in a SLA1500 rotor for 1 hour at 4^o^C to obtain the membrane-enriched P fraction. The P fraction was washed with 250ml CSB and suspended in 200ml membrane extraction buffer, MEB [20mM Phosphate, (pH7.2)/150mM NaCl/5mM EDTA/2% Tween 20/4M urea] and extracted for 2 hours at RT. The solubilized P fraction was clarified by centrifugation as above, diluted with an equal volume of MEB lacking Tween 20 and urea, chilled to 4^o^C and subjected to polyethylene glycol (PEG) precipitation as follows. Forty ml of a 50% aqueous solution of PEG 6000 were added gradually over ~30 minutes (7-8ml/5 minutes) with continuous stirring. The contents were left stirring overnight at 4^o^C, and clarified by centrifugation (SLA 1500 rotor as above) and filtration (0.45µ). This was subjected to tangential flow filtration (TFF) across a 300 kDa cut-off membrane using 4 liters each of 2M, 1M and 0M urea in TFF buffer [20mM Phosphate (pH 7.2)/150mM NaCl/5mM EDTA]. The retentate (~400ml) obtained at this point was filtered (0.45µ) and bound (for 2 hours at 25^o^C in a shaker at 200 rpm) to 30ml Phenyl 600M Toyopearl resin (pre-equilibrated in TFF buffer without urea) in batch mode. The resin was packed into a 50ml Sigma chromatographic column and washed with 5 bed volumes of TFF buffer without urea, followed by a linear decreasing salt gradient (150 to 0mM NaCl) in the same buffer, over 2 bed volumes. After reaching 0mM NaCl, washing was continued for a further 3 bed volumes. Bound proteins were eluted using a 0-8M urea step gradient (with 2M increase at each step lasting 5 bed volumes) in 20mM sodium bicarbonate buffer (pH 9.6). Column fractions were analysed by SDS-PAGE, purified peak fractions pooled, and dialyzed against 1× PBS.

**1.5 Characterization**

ES protein was detected using sandwich ELISA in two formats. In the first format, microtiter wells were coated with DENV-2 EDIII-specific mAb 24A12 (500ng/100µl) in sodium bicarbonate buffer (pH 9.6) overnight at 4^o^C, washed with 1× PBS/0.5% Tween 20, and blocked with 1× PBS/5% skim milk/2% polyvinyl pyrrolidone (300µl/well). Wells were washed again followed by the addition of 100µl of either S- (diluted 1:250 using 1× PBS/0.5% skim milk) or P- (diluted 1:500) fractions obtained from induced cells. This was incubated at 37^o^C for 1 hour, washed with 1×PBS/0.5% Tween 20 (5×) and incubated 1 hour at 37^o^C with 50µl anti-S-mAb-HRPO (Hepanostika) per well. Wells were washed again and treated with TMB substrate (50µl/well). After color development (37^o^C, 10 minutes), the reaction was stopped with 1N H_2_SO_4_ and absorbance read at 450 nm. In the second sandwich format, both antibodies were S antigen-specific and were from the Hepanostika kit and the assay was performed as per the manufacturer’s directions. In this latter assay, S- and P-fraction dilutions were 1:250 and 1:2000, respectively.

Immunoblot analyses were performed essentially as reported earlier (*Arora et al, 2012; Patil & Khanna, 2012*). Briefly, after denaturing gel electrophoresis (SDS-15% polyacrylamide) and electro-transfer (12V, 30 minutes), the nitrocellulose membrane was blocked (5% skim milk/1× PBS/0.1% Tween 20, 2 hours at RT) rinsed (1× PBS/1% Tween 20) and probed either with EDIII-specific mAb 24A12 (1μg/ml) or 5S mAb (1μg/ml). Blots were washed and developed using anti-mouse IgG-HRPO conjugate plus TMB substrate. To assess the relative abundance of the antigen bands detected in the blots, densitometric image analysis was performed. This was done using the NIH-developed public domain Java image processing program, ImageJ, (<http://rsb.info.nih.gov/ij/>), according to the developer’s guidelines.

The presence of higher order structures in partially purified preparations was assessed by CsCl gradient analysis as described (*Vassileva et al, 2001*). Briefly, aliquots of the sample were layered on a 20% CsCl gradient and centrifuged at 45,000 rpm (SW60 rotor, 45,000 rpm, 16 hours, 20^o^C), followed by collection of fractions from the bottom of the gradient. Fractions were analysed using sandwich ELISAs (below).

The presence of virus-like particles (VLPs) in the purified preparations was visualized by electron microscopy as before (*Arora et al, 2012*). Formvar carbon-coated grids were soaked in the purified material, which was diluted to ~5-10µg/ml for ~5 minutes. The grids were blotted with Whatmann paper to remove excess solution and soaked next in 1% uranyl acetate for 2 minutes, blot-dried once again and examined under a Tecnai electron microscope.

**1.6 Functional analysis**

Competitive ELISA was done essentially as reported (*Arora et al, 2012*). Briefly, 100μl aliquots of mAb 24A12 (10ng/ml) were pre-incubated (1 hour at 37^o^C) with equal volumes of purified ES,S_0_, ES,S_1_, ES,S_2_ and ES,S_4_ preparations (ranging from 1-3μg/ml) and added to microtiter wells (100μl/well) coated with yeast-expressed EDIII-2 (*Batra et al, 2010*). Controls wherein the mAb was mock-pre-incubated with 1× PBS were run in parallel. Wells were washed with 1× PBS/0.1% Tween 20, and incubated with anti-mouse IgG-HRPO conjugate (0.1μg/ml; 100μl/well) for 1 hour at 37^o^C. This was followed by color development and absorbance measurement as above.

Binding blocking assay was performed as follows. Vero cells were seeded in 96-well plates at 35,000 cells in 100µl/well [in DMEM+5% heat-inactivated (Δ) fetal bovine serum (FBS)] and incubated at 37^o^C in a 10% CO_2_ incubator. At 24 hours post-seeding, medium was aspirated from the wells followed by addition of 100µl VLP (0.2µg, in DMEM+2% Δ FBS) and incubated at 37^o^C. After 1 hour exposure to VLPs, the wells were aspirated, and the cells infected with DENV-2 (250 plaque forming units/100 µl/well in DMEM+2% Δ FBS). About 2 hours later, the virus inoculum was removed from the wells followed by the addition of 200µl DMEM+5% Δ FBS/well. Cells were incubated for 8 days. Culture supernatants were sampled at daily intervals for release of viral NS1 antigen using Dengue NS1 Platelia kit as per the manufacturer’s directions (*Korrapati et al, 2012*).


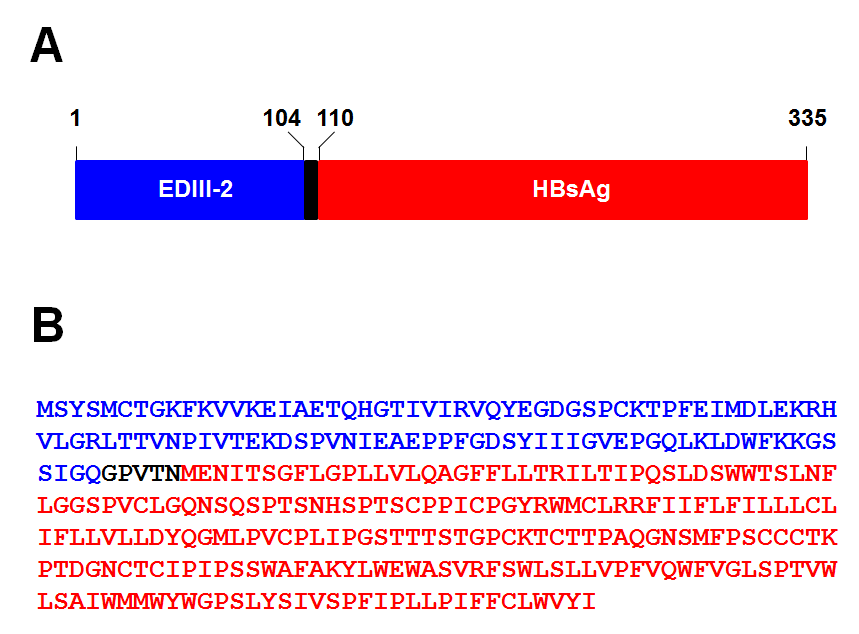
**2. SUPPLEMENTARY FIGURES**

**Figure S1: The design and sequence of the ES fusion antigen.** (A) Schematic representation of the ES fusion antigen consisting of a 104 aa residue long DENV-2 EDIII polypeptide (blue) linked to a 226 aa residue long HBsAg polypeptide (red) through a pentapeptide linker (black). (B) The complete aa sequence of the ES antigen. The color scheme corresponds to that shown in ‘A’.


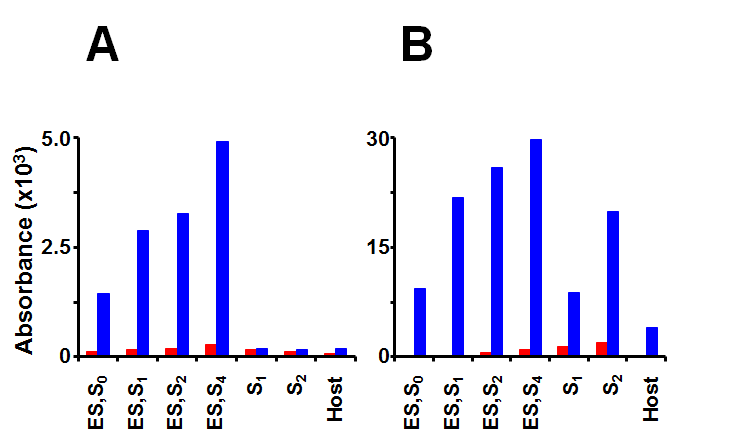


**Figure S2: Localization and extraction of ES,S antigens.** (A) Methanol-induced *P. pastoris* clones harbouring *ES* genes in the background of 0 (ES,S_0_), 1 (ES,S_1_), 2 (ES,S_2_) and 4 (ES,S_4_) copies of the *S* gene, were lysed and separated into S and P fractions. The presence of the ES antigen in the S (red bars) and P (blue bars) fractions was detected in a sandwich ELISA using mAb 24A12 as the coating antibody and anti-HBs-HRPO conjugate (from Hepanostika kit) as the revealing antibody. S_1_ and S_2_ denote *P. pastoris* clones harbouring 1 and 2 copies, respectively, of the *S* gene alone; ‘Host’ denotes the parent *P. pastoris* strain which does not carry either the *ES* or *S* genes. (B) The experiment in panel A was repeated using Hepanostika kit wherein both capture and reveal antibodies are specific to the S antigen. Experiments in panels A and B were performed twice; one experiment each is shown.


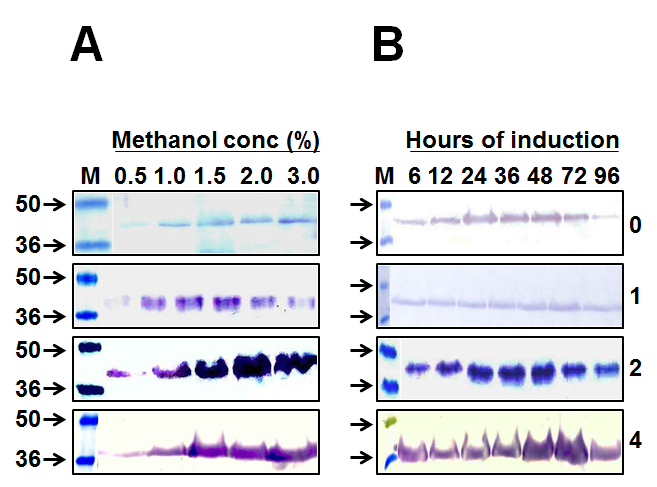


**Figure S3: Optimization of induction of the different ES,S antigens.** *P. pastoris* clones harbouring the *ES* fusion genes in the background of different *S* gene copy numbers, were induced either at varying concentrations of methanol (ranging from 0.5-3%) for a constant period of time (48 hours, panel A) or at a fixed methanol concentration (1 %) for varying durations (6-96 hours, panel B), followed by detection of the expressed ES antigen by immunblotting with mAb 24A12. For simplicity, only the relevant portions of the blots are shown. In both panels, pre-stained protein markers were run in lanes marked ‘M’, with the arrows indicating the positions of individual markers; their sizes (in kDa) are shown to the left of panel ‘A’. The *S* gene copy number of the clones tested is indicated by the Arabic numerals, 0-4, to the right of panel B.


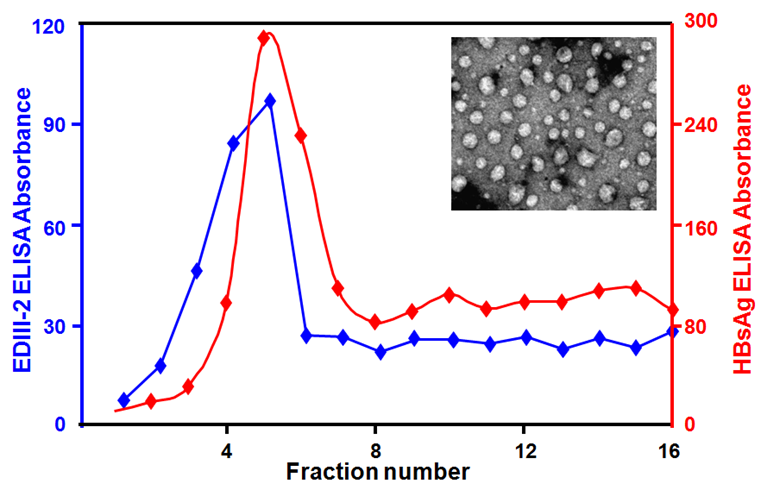
**Figure S4: CsCl sedimentation analysis of *P. pastoris*-expressed ES,S_4_ antigen.** A partially purified preparation of the ES,S_4_ antigen was sedimented down a CsCl gradient. Fractions were collected sequentially from the bottom (fractions 1 and 16 represent the bottom-most and top-most fractions, respectively) and analyzed by ELISA using either EDIII-specific mAb (blue curve) or HBsAg-specific mAb (red curve) to capture the recombinant antigen. In both cases the revealing antibody was a separate HBsAg-specific mAb. The inset shows an electron micrograph of the peak fraction (fraction #5).

**3. REFERENCES**

- Arora U, Tyagi P, Swaminathan S, Khanna N. Dengue virus type 2 envelope domain III displaying bio-nanoparticles. *J Nanobiotechnol* 2012; **10:** 30.
- Batra G, Raut R, Dahiya S, Kamran N, Swaminathan S, Khanna N. *Pichia pastoris*-expressed dengue virus type 2 envelope domain III elicits virus-neutralizing antibodies. *J Virol Methods* 2010; 167: 10-16.
- Khanam S, Pilankatta R, Khanna N, Swaminathan S. An adenovirus type 5 (AdV5) vector encoding an envelope domain III-based tetravalent antigen elicits immune responses against all four dengue viruses in the presence of prior AdV5 immunity. *Vaccine* 2009; 27: 6011-6021.
- Korrapati AB, Swaminathan G, Singh A, Khanna N, Swaminathan S. Adenovirus delivered short hairpin RNA targeting a conserved site in the 5’ non-translated region inhibits all four serotypes of dengue viruses. *PLoS Negl Trop Dis* 2012; 6: e1735.
- Patil A, Khanna N. Novel membrane extraction procedure for the purification of hepatitis B surface antigen from *Pichia pastoris*. *J Chromatog B* 2012; 898: 7-14.
- Vassileva A, Chugh DA, Swaminathan S, Khanna N. Effect of copy number on the expression levels of Hepatitis B surface antigen in the methylotrophic yeast *Pichia pastoris*. *Protein Exp Purif* 2001; 21: 71–80.

***
